# Completing the hierarchy of rotational defects in monolayer MoS_2_ through symmetry-aware evolutionary search

**DOI:** 10.1039/d5cp03121d

**Published:** 2025-12-18

**Authors:** Alexander Adel, Ralf Wanzenböck, Georg K. H. Madsen

**Affiliations:** a Institute of Materials Chemistry, TU Wien A-1060 Vienna Austria georg.madsen@tuwien.ac.at

## Abstract

Monolayer molybdenum disulfide (MoS_2_) shows a plethora of defect configurations, which constitutes the basis for tailoring material properties through defect engineering. Detailed characterization of these defects remains challenging due to the complexity of the potential energy surface. We efficiently explore the three-fold rotational defect potential energy surface in monolayer MoS_2_ by combining an evolutionary algorithm with a machine-learning force field. To improve the performance of the structure searches, the algorithm hierarchically restricts the exploration process to a lower-dimensional subspace, utilizing the symmetry operators associated with the investigated defects. We demonstrate that these constrained trajectories exhibit lower global uncertainty measures during the evolution, produce final structures with lower energy distributions and converge faster. Our approach results in the discovery of several novel structures with reasonable computational effort, thereby completing the hierarchy of rotational defects in MoS_2_.

## Abstract 2

This work combines a symmetry-constrained evolutionary algorithm with a machine-learning force field backend to discover new three-fold rotational defects in monolayer molybdenum disulfide (MoS_2_).

## Graphical abstract

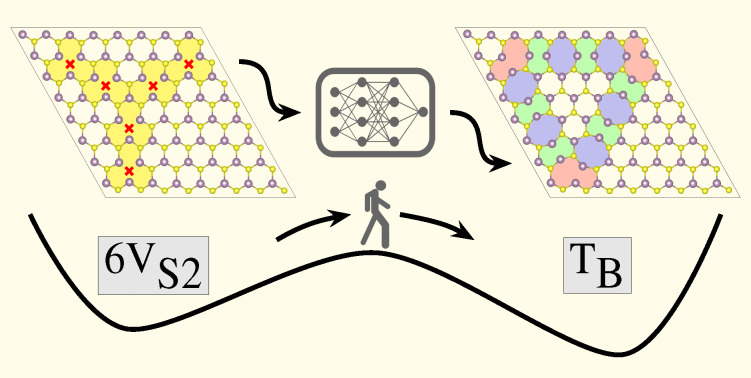

## Introduction

As one of the most prominent members of the transition-metal-chalcogenides family, molybdenum disulfide (MoS_2_) features extensive studies regarding its layered structure, from the preparation of very thin crystals^1–3^ to the isolation of individual crystal planes.^4^ Monolayer MoS_2_ consists of three atomic layers of alternating Mo and S. Notably, this two-dimensional material is a direct band gap semiconductor.^5–7^ Monolayer MoS_2_ shows a plethora of defect configurations. The size and complexity of these configurations range from small native point defects such as vacancies, antisites, adatoms, and interstitials^8–10^ to large structural disturbances such as extended line defects^11–13^ and grain boundaries.^14,15^ This large variety constitutes the basis for the manipulation of material properties and defect engineering.^16–21^

A specific type of defect in MoS_2_ are the topological defects obtained when six S atoms (three in the upper and three in the lower atomic layer) are rotated by 60° around an Mo atom in the central layer.^22^ Parallel to how the well-known Stone–Wales defects in graphene^23^ can order in extended patterns,^24^ the rotational MoS_2_ defects, in conjunction with sulfur double vacancies, have been found to produce quite extensive reconstructions, dependent on the number of these transformations occurring in the vicinity of each other.^22^ As the defect structures grow in size, so does the complexity of the energy landscape^25^ and finding the stable structures by constructing viable low-energy defect configurations according to domain knowledge becomes untenable.

Evolution strategies are a frequently used approach for rugged energy landscapes.^26–28^ To enhance the efficiency of global atomistic structure optimizations, search algorithms can exploit the inherent symmetries of the system under investigation. One approach is to bias the potential energy surface directly,^29^ but a conceptually simpler approach is to restrict the search space to configurations that obey predefined symmetry constraints.^30–34^ This reduction in the number of degrees of freedom narrows the configurational space and allows the algorithm to identify plausible structures more efficiently.

In general, stochastic approaches like evolution strategies still require a substantial volume of fitness evaluations. To make the calculations feasible, highly parametrized models based on machine learning have been used.^35–37^ In this paper, we show that the utilization of machine-learning force fields (MLFF), combined with the systematic reduction of the degrees of freedom using symmetry, makes the efficient treatment of complex defect structures possible. Specifically, we apply our constrained evolutionary algorithm to investigate the aforementioned large-scale rotational defects in monolayer MoS_2_.

The paper is organized as follows. We start with a description of the process of the constrained structure search and the generation of the data set required for the MLFF training. Next, we discuss the characteristics of the constrained trajectories based on the predicted energies and force uncertainties obtained by the MLFF. This is followed by a presentation of the resulting structures in the rotational defect hierarchy. Finally, the MLFF is also applied to monolayer MoS_2_ grain boundaries. The paper ends with a conclusion and an outlook.

## Methods

### DFT calculations

The reference values for the data sets were obtained by performing spin-polarized calculations with the VASP^38,39^ implementation of the projector augmented-wave formalism,^40^ using the Perdew–Burke–Ernzerhof (PBE) functional.^41^ The energy cutoff was set to 258 eV (the default value for sulfur) and the break condition for the electronic self-consistent loop to 10^−6^ eV. Only the Γ-point was used for the defected supercell and the width of Gaussian smearing was set to 10^−4^ eV. All supercells were set up with one monolayer separated from the vertical borders of the supercell by vacuum with a thickness of 22 Å. Convergence tests of the energy cutoff and *k*-mesh were performed by comparing the energy difference of the most stable structures found with symmetry constraint *C*_3_ + *σ*_h_ and with symmetry constraint *σ*_h_. The energy difference was found to change by less than 0.01 eV compared to a total energy difference of 1.17 eV.

### Structure search

We utilize the covariance matrix adaptation evolution strategy (CMA-ES)^28^ as implemented and tailored to atomistic structure searches in Clinamen2.^42^ The Clinamen2 code was extended by a module implementing symmetry operations such as rotations and reflections. Before the random sampling step takes place, all individual structures of the current population are reduced to the asymmetric unit corresponding to the selected symmetry constraint. After sampling, the structures are rebuilt so that the energy evaluation step is performed for the complete structures.

### Symmetry operations

**Fig. 1:**
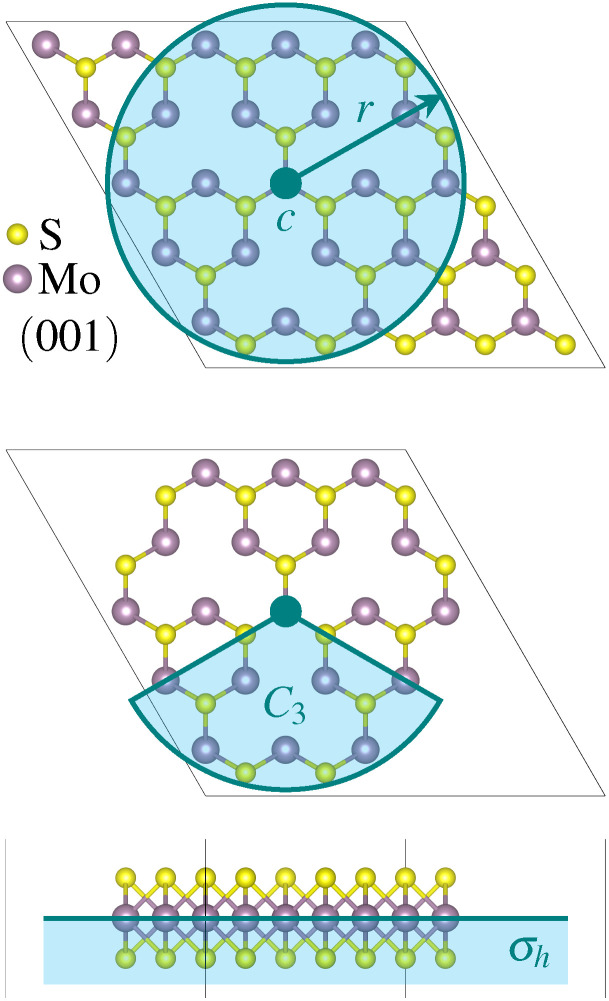
Overview of the applied symmetry constraints. Top view of a monolayer MoS_2_ founder structure with three sulfur double vacancies (3V_S_2__) inside a (5 × 5 × 1) supercell. The atoms modified during the evolution are placed inside a sphere defined by radius *r* around the center *c*. The atoms outside the sphere are fixed. This setup corresponds to the baseline none (*C*_1_). Further reduction of the number of degrees of freedom is achieved by applying the threefold (*C*_3_) rotational axis and the horizontal (*σ*_h_) mirror plane.

The relevant point group of the three-fold rotational defects is *D*_3h_. We considered four symmetry constraints, none (*C*_1_) as a baseline, a horizontal mirror plane (*σ*_h_), a threefold rotation axis perpendicular to the MoS_2_ plane (*C*_3_), and the combination of *C*_3_ and *σ*_h_. The two symmetry operators are illustrated in Fig. 1, together with the initial or founder structure used for the smallest defect.

### 3V_S_2__ data set generation

The data set for MoS_2_ structures with three sulfur double vacancies inside a (5 × 5 × 1) supercell (symbolized by 3V_S_2__) was constructed as follows: for each combination of symmetry operations, one Clinamen2 evolution (with Vasp as evaluation back-end) was performed for 100 generations (with *σ*^init^ = 0.40 Å and population sizes between 12 and 18, dependent on the symmetry). Non-physical structures were filtered out by discarding all data points that contained at least one force component larger than a specific value (between 100 and 240 meV Å^−1^, dependent on the symmetry) and/or had an energy value larger than 0.0 eV. To decrease the redundancy of the data set, it was further reduced by selecting only five structures per generation (highest energy, lowest energy, and additional three randomly chosen) for further treatment. To clean the data set from structures that do not represent two-dimensional materials, all structures that contained at least one atom with a distance larger than two Mo–S bond lengths from the horizontal monolayer plane were also removed. In the end, the split consisted of 921 structures for the training set (50%), 180 structures for the validation set (10%), 373 structures for the active learning sample set (20%) and 373 structures for the test set (20%), in total 1847 structures.

### Machine-learning force fields

The 3V_S_2__ data set (described above) was used for the training of the MACE^43^ MLFF. The hidden layers were set to 64 channels and the cutoff radius to 4 Å. In the first phase of training (maximum 1200 epochs), the energy and force weights were set to 1 and 100, respectively. In the second phase (maximum 300 epochs), the weight for the energy was increased to 1000. A second MLFF, based on the NeuralIL^44,45^ architecture, was used only for the generation of the 5V_S_2__ test set (described below). The training fraction was set to 0.8, the cutoff radius to 4 Å, the number of basis functions to 5, the energy weight to 0.5 and the number of epochs to 51. The ResNet core widths were chosen as 64, 32 and 16.

### 5V_S_2__ test set generation

To generate the test set of MoS_2_ structures with five sulfur double vacancies inside a (7 × 7 × 1) supercell (symbolized by 5V_S_2__), the following steps were performed: for each symmetry operation, the 100 structures with the highest uncertainties, see eqn (1), were selected from 10 Clinamen2 evolutions. These were executed with *σ*^init^ = 0.40 Å, different random seeds, and the NeuralIL MLFF as evaluation back-end. The same procedure was repeated with additional 10 evolutions, but now with *σ*^init^ = 0.60 Å. The removal of high force, high energy, and non-two-dimensional structures (similar to the 3V_S_2__ data set) resulted in a test set consisting of 496 structures in total.

### Uncertainties

The force uncertainties were estimated by training two committees (MACE and NeuralIL) consisting of 5 models each. The only difference between the models of a committee is the random seed that determines the initial weights of the neural network. Every model in the committee produces their own predictions for the force components of the atoms. The individual force uncertainties were aggregated over all atoms in a given configuration to the global uncertainties1
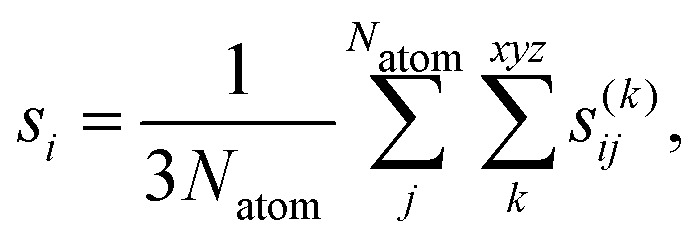
with *k* denoting the spatial direction, *j* the atom, and *i* the configuration. These uncertainties have been shown to provide reliable estimates of the error, exhibiting a strong correlation with the corresponding differences between the predicted and reference DFT values.^45–47^

### Active learning

For the active learning step, the structures from the 3V_S_2__ active learning sample set were sorted by their uncertainty as defined in eqn (1). The 74 structures in the highest 20% interval were removed from the active learning sample set and instead added to the training and validation sets. The resulting data set represents iteration 1 (see Fig. S2 in the SI).

## Results

### Force field predictions

**Fig. 2:**
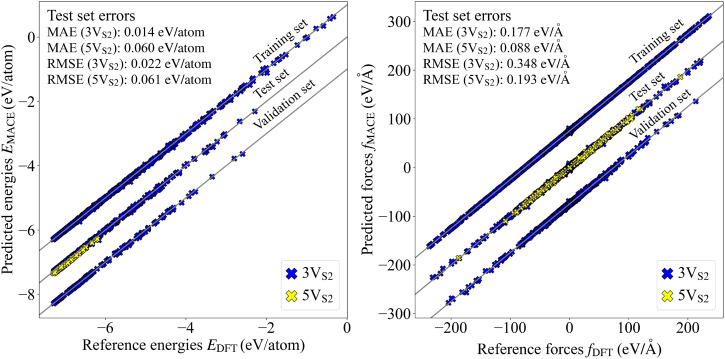
Parity plots for the predicted and reference energies and forces. The plots compare the predicted energies per atom *E*_MACE_ and forces *f*_MACE_ from the MLFF with reference energies per atom *E*_DFT_ and forces *f*_DFT_ calculated by DFT. Shown are all three 3V_S_2__ data set splits, where the training and validation set lines are shifted up and down relative to the test set line, respectively. The 5V_S_2__ data set is only used as a test set. Also given are the test set errors (MAE and RMSE) for both types of founder structures.

The data set for the MACE MLFF committee contained structures only from evolutions that started with the smallest founder, consisting of a single layer of MoS_2_ with three double vacancies (3V_S_2__). Additionally, a test set including larger founders with five double vacancies (5V_S_2__) was generated. To measure the performance of the MLFF, parity plots for both 3V_S_2__ and 5V_S_2__ data sets were generated, see Fig. 2. The error values demonstrate the predictive power of the force field for the 3V_S_2__ structures and the ability to generalize well to the larger 5V_S_2__ structures, which were not part of the training set.

**Fig. 3:**
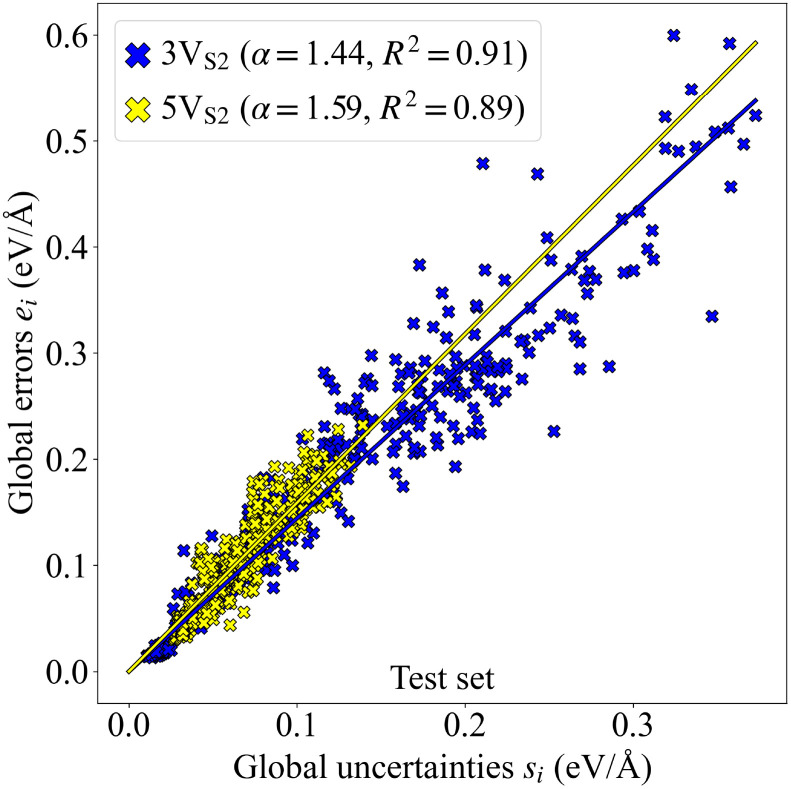
Alpha correlation plot. The global errors *e*_*i*_ (the difference between the predicted and the reference forces) are plotted over the global uncertainties *s*_*i*_ resulting from the force predictions of the MLFF committee. Linear fits for both 3V_S_2__ and 5V_S_2__ data sets are visualized by colored lines. Additional characteristics of the linear fits such as the slope *α* and the coefficient of determination *R*^2^ are given in the legend.

The correlation between the uncertainties and error values is visualized in a correlation plot, Fig. 3, where also linear fits of the data points are shown. The value of the slope *α* of the linear fit for the 3V_S_2__ structures is smaller than for the 5V_S_2__ test set. Since higher *α* values are generally related to higher model bias,^47^ it seems plausible to receive a higher *α* value when the force field has to extrapolate the prediction to larger, previously unseen structures. Nevertheless, the difference is small, and the uncertainties can, therefore, be used as a reliable measure for both founder types to describe the performance of the MLFF during the executed evolutions.

The global uncertainties classify all obtained structures into two categories. The first contains configurations which are familiar to the force field (low uncertainty) and the second contains configurations where the force field is unsure if the predictions are accurate (high uncertainty). This classification more or less decides which structures contain the most information that could be useful for the improvement of the MLFF. Keeping this principle in mind, active learning steps can be performed, where the structures with the highest uncertainties are selected and added to the training set for a new force field. In our case, the top 20% 3V_S_2__ structures exhibiting the largest uncertainty were removed from the active learning sample set and instead added to the training and validation sets. Then a new MLFF was trained on these augmented data sets. In the following, the original force field before the active learning step will be called iteration 0, while the new force field after the active learning step will be called iteration 1. Parity and alpha correlation plots were generated to compare the predictive power between these two MLFFs (see Fig. S2 in the SI). The test set errors contained in these plots are summarized in Table 1. The results indicate that the active learning step does not show a large improvement regarding the predictive power. The energy and force errors for both the 3V_S_2__ and 5V_S_2__ structures only changed minutely. We therefore settled on the MLFF trained purely on the 3V_S_2__ structures obtained from the original CMA-ES evolutions.

Test set errors for one active learning step. Test set errors for the MLFF before (iteration 0) and after (iteration 1) one active learning step. Given are the MAE and RMSE for the predicted energies per atom *E*_MACE_ and the predicted forces *f*_MACE_ for both 3V_S_2__ and 5V_S_2__ data sets

**Table:** 

*E*_MACE_ (eV per atom)	3V_S_2__		5V_S_2__	
	MAE	RMSE	MAE	RMSE
Iteration 0	0.014	0.022	0.060	0.061
Iteration 1	0.013	0.021	0.057	0.058

**Table:** 

*f*_MACE_ (eV Å^−1^)	MAE	RMSE	MAE	RMSE
Iteration 0	0.177	0.348	0.088	0.193
Iteration 1	0.175	0.342	0.087	0.189

### Constrained trajectories

**Fig. 4:**
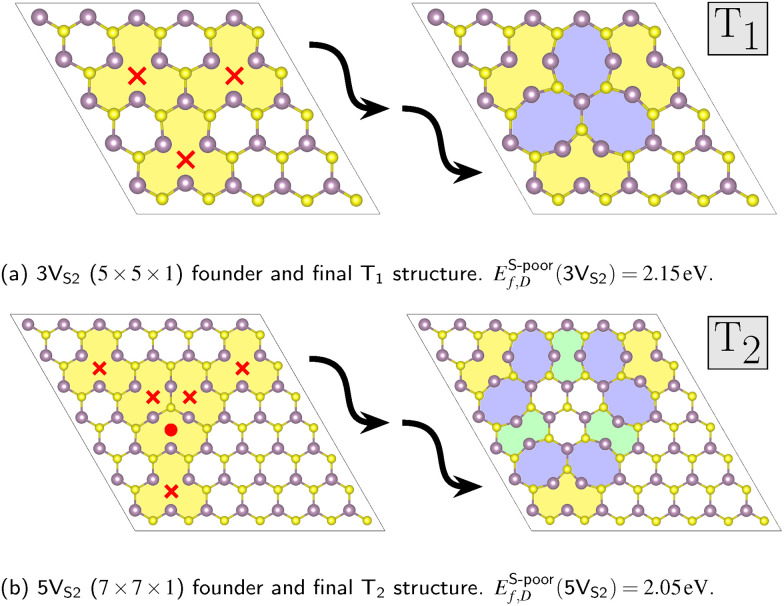
Founders with double vacancies and final structures with rotational defects. The founders are placed in differently sized super cells (*D* × *D* × 1): (a) D = 5 and (b) D = 7. Both contain sulfur double vacancies *X*V_S_2__ with *X* ∈ {3,5}, respectively. The double vacancies are symbolized by red crosses. Notice that in the case of 5V_S_2__, one pair of sulfurs is not removed (shown as a red dot), but shifted to the center to preserve the rotational symmetry. The final structures, produced by *C*_3_ + *σ*_h_ constrained evolutions, are colored to highlight the octagons (blue), double-pentagons (green), and ten-fold rings (yellow) of the defects. Also given are the defect formation energies *E*_f,D_ = *X*^−1^(*E*_D_ − *E*_bulk_ − *n*_*α*_*E*_*α*_), normalized by the number of double vacancies *X*.

Three-fold rotational defects can be ordered into a hierarchy, where the geometric properties of each level, labeled as T_*n*_ with *n* ∈ {1, 2, 3,…}, can be described by structural parameters. One example would be the number of octagons and double-pentagons which are part of the defect.^22^ The requirement for the formation of these defects is a specific number of sulfur double vacancies in the vicinity of potential bond rotation centers. The smallest defect, called T_1_, requires three double vacancies (3V_S_2__) around one rotation center (see Fig. 4a). The vacancies in the founder were placed symmetrically around the center, since the search algorithm had to start the evolutions with the same symmetry constraints as the desired final structure. This was also the reason why one of the sulfur pairs of the founder of the defect level T_2_, which requires five double vacancies (5V_S_2__), was shifted to the intersection between the rotational axis and the horizontal monolayer plane (see Fig. 4b).

**Fig. 5:**
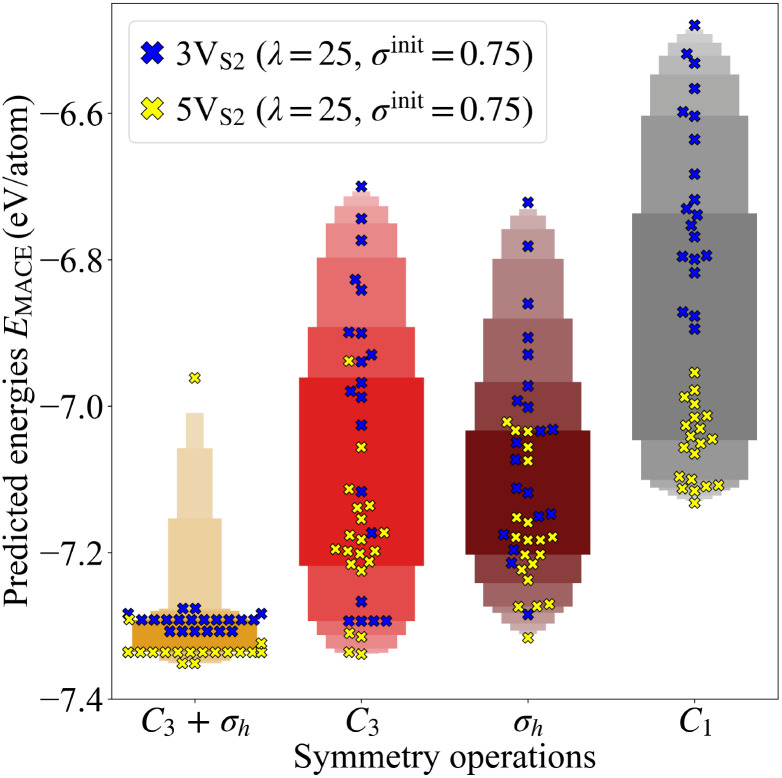
Predicted energy per atom distributions for all symmetry operations. Shown are the predicted energies per atom *E*_MACE_ for the final structures from in total 160 evolutions. Both 3V_S_2__ and 5V_S_2__ founders were included. The population size was selected as *λ* = 25 and the initial step size set to *σ*^init^ = 0.75 Å. The utilized constraints are ordered from the highest symmetry (*C*_3_ + *σ*_h_) on the left to the lowest symmetry (*C*_1_) on the right. The shading in the background indicates the accumulation area around the mean value of the distributions.

To demonstrate the influence of the applied constraints, we ran 20 separate Clinamen2 evolutions with different random seeds and the MACE MLFF as evaluation back-end for each of the four symmetry operations. This was done for both the smaller 3V_S_2__ and the larger 5V_S_2__ founders, thus yielding 160 CMA-ES evolutions in total. The population size was selected as *λ* = 25, while the initial step size was chosen as *σ*^init^ = 0.75 Å. The advantages of these constrained evolutions can be shown by analyzing the energy per atom distributions of the final structures of the trajectories. For every evolution, the predicted energy per atom of the most stable structure in the population of the last generation was plotted, see Fig. 5. The distribution of both founder types shows that evolutions with symmetry constraints lead to lower average values of the energies per atom of the final structures. Out of all 3V_S_2__ evolutions (20 runs) with the *C*_3_ + *σ*_h_ constraint, the search found the smallest of the rotational defects, T_1_ in Fig. 4a, six times. Out of all 5V_S_2__ evolutions run with the same constraint (also 20 runs), the larger T_2_ defect was found two times (see Fig. 4b). Both these structures are in excellent agreement with the combined DFT and experimental study in ref. 22, underlining the significant promise of the transferable MLFF. Another important benefit shown by Fig. 5 is the fact that evolutions augmented with symmetry constrains permit both lower population sizes *λ* and higher initial step sizes *σ*^init^, while still producing stable final structures at the same time. Low population sizes lead to faster execution times, while high initial step sizes increase the possibility of the evolution mean to leave the minimum of the founder, opening up the opportunity to find other stable minima. Additional energy per atom distributions for other initial step and population sizes can be found in Fig. S3 in the SI.

**Fig. 6:**
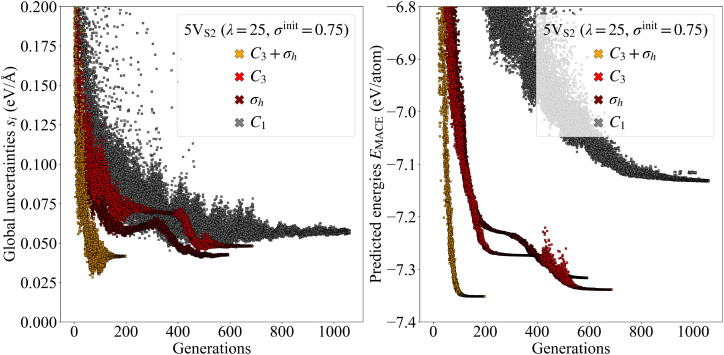
Uncertainties and predicted energies per atom plotted over the number of generations. The left figure shows the global uncertainties *s*_*i*_ of all structures produced by selected evolutions as a function of the generations. For every symmetry operation, one evolution is plotted, coded by color. All evolutions started with the 5V_S_2__ founder. The population size was selected as *λ* = 25 and the initial step size set to *σ*^init^ = 0.75 Å. The right figure plots the predicted energies per atom *E*_MACE_ of the same evolutions over the generations.

Another way to visualize the usefulness of our symmetry constraints is to plot the calculated uncertainties of all structures as a function of the number of generations, see Fig. 6. The uncertainty trajectories were extracted from the four 5V_S_2__ evolutions that led to the final structures with the lowest energies out of all evolutions. The plot shows that constrained trajectories, in general, exhibit global uncertainties with a lower mean and a smaller standard deviation per generation than completely free trajectories. In addition, on average, the termination criteria are satisfied earlier, which leads to faster convergence. Furthermore, the final structures produced by these high-symmetry evolutions possess lower predicted energy per atom distributions, leading to more stable configurations.

To substantiate the claim of a symmetry-related advantage, we performed a series of DFT calculations for selected final structures shown in Fig. 5. The deviations in Δ*E* between the MACE model and DFT are provided in Table S1 in the SI and fall within the range expected from the model's MAE and RMSE (Fig. 2). Moreover, the DFT energy differences between the final structures corroborate the stability ordering predicted by the force field.

The dynamic change of the uncertainty values during the evolutions offers insight into different phases of the search process. For example, the trajectory for the evolution with the *C*_3_ constraint (see Fig. 6) experiences an interesting sequence of character shifts. Approaching generation 300, the spread of the uncertainties is already very slim and decreases even further, which normally indicates the end of an evolution. However, around generation 400, the standard deviation increases sharply, suggesting that the CMA-ES found a new path for further exploration. The same characteristics can be seen for the energies. The evolution is following this track until around generation 500, during which both the uncertainties and the energies are decreasing. Finally, around generation 600, the search converges to the stable configuration, while at the same time scaling back once again to small standard deviations for both quantities.

### Structure search

**Fig. 7:**
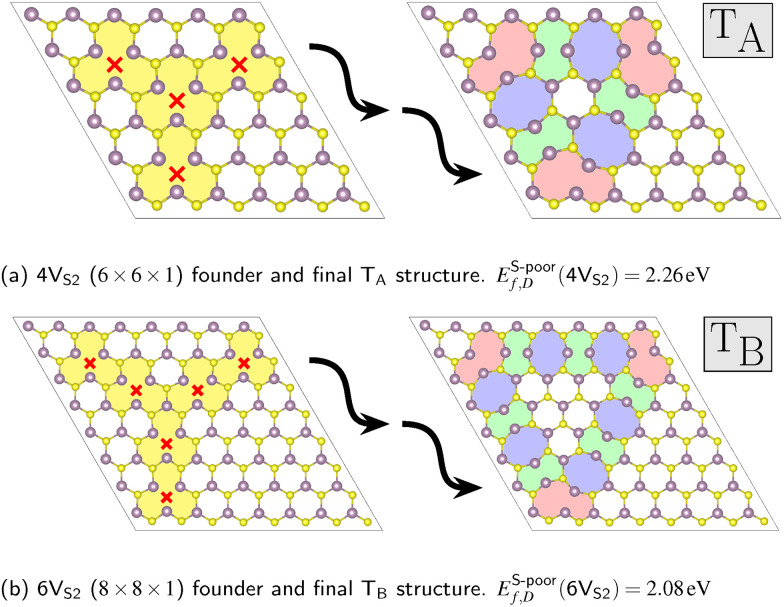
Founders and final structures with new rotational defects from the structure search. The founders are placed in differently sized super cells (*D* × *D* × 1): (a) *D* = 6 and (b) *D* = 8. Both contain sulfur double vacancies *X*V_S_2__ with *X* ∈ {4,6}, respectively. The coloring scheme follows Fig. 4. The corners of the T_A_ and T_B_ structures colored in red consist of one octagon and one pentagon each.

The MLFF was not only able to reproduce the two known members of the hierarchy, Fig. 4, but also capable to find additional rotational defects. These require an amount of double vacancies between the numbers already presented, specifically 4V_S_2__ and 6V_S_2__, see Fig. 7a and b. These defects contain the same structural 8-5-5-8 ring building blocks as the known structures in Fig. 4. They also exhibit corners consisting of one octagon and one pentagon each (red colored areas in Fig. 7a and b). These T_A_ and T_B_ defect structures fill the gaps in the defect hierarchy, completing the series ranging from 3V_S_2__ to 6V_S_2__ founders.

**Fig. 8:**
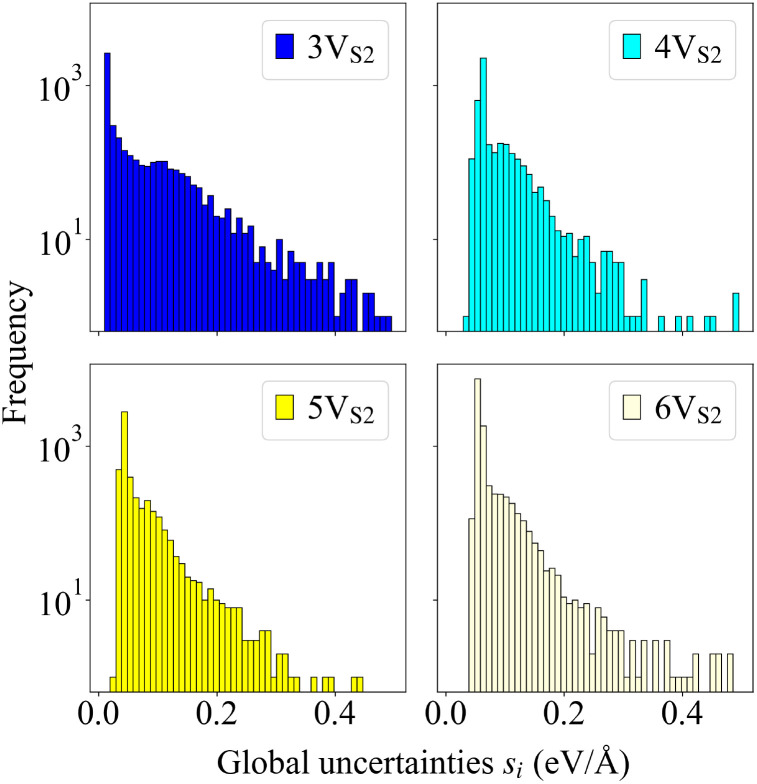
Histograms of global uncertainties. Shown are the frequency distributions of the global uncertainties *s*_*i*_ for all four evolutions that generated the final structures illustrated in Fig. 4 and 7, visualized as histograms. Uncertainties larger than *s*_*i*_ = 0.5 eV Å^−1^ are not shown, since these values are very low in number and arise only at the very beginning of the evolutions.

Although the force field was trained on structures that only contained small T_1_ defects, it was able to generalize well to predictions of larger T_2_ structures and to extrapolate from the existing data to T_A_ and T_B_ defects. Fig. 8 indicates that the global uncertainties are still a reliable measure of performance when utilized for the new 4V_S_2__ and 6V_S_2__ founders, since the visual comparison shows frequency distributions similar to those started with the 3V_S_2__ and 5V_S_2__ structures.

As a final validation of the search procedure, we initiated new evolutionary runs using the high-symmetry structures as founders, while enforcing lower symmetry constraints. The low-energy threefold-rotational defects shown in Fig. 4 and 7 were used as starting configurations for evolutions constrained by *C*_3_, *σ*_h_, and *C*_1_ symmetries. In all cases, the evolutions converged to the same defect structures, thereby reinforcing the conclusion that the rotational defects correspond to genuine local minima.

### Grain boundaries

The MLFF was trained on only 1101 structures from the original DFT CMA-ES trajectories of small 3V_S_2__ defects, but showed the ability to generalize to structures not included in training. To further demonstrate this feature, it was applied to MoS_2_ monolayer grain boundaries. These geometries contain similar building blocks as the rotational defects discussed above.^14^ Specifically, we used the MACE committee to calculate the local uncertainties2
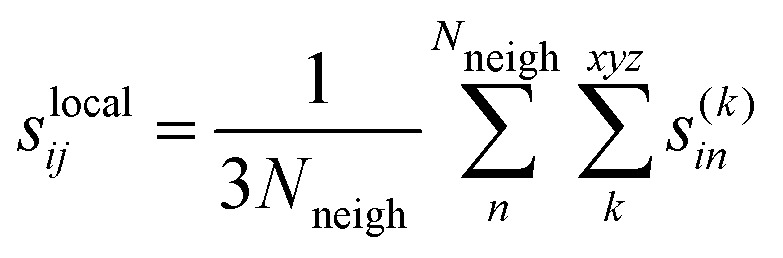
for every atom in the vicinity of a grain boundary with a tilt angle of 18.5°. *N*_neigh_ denotes here the neighbouring atoms of atom *j*, located within a cutoff radius *r*_cut_. These locally aggregated uncertainties have been shown to correlate with the local errors in a similar way as the globally aggregated uncertainties in Fig. 3.^47^

**Fig. 9:**
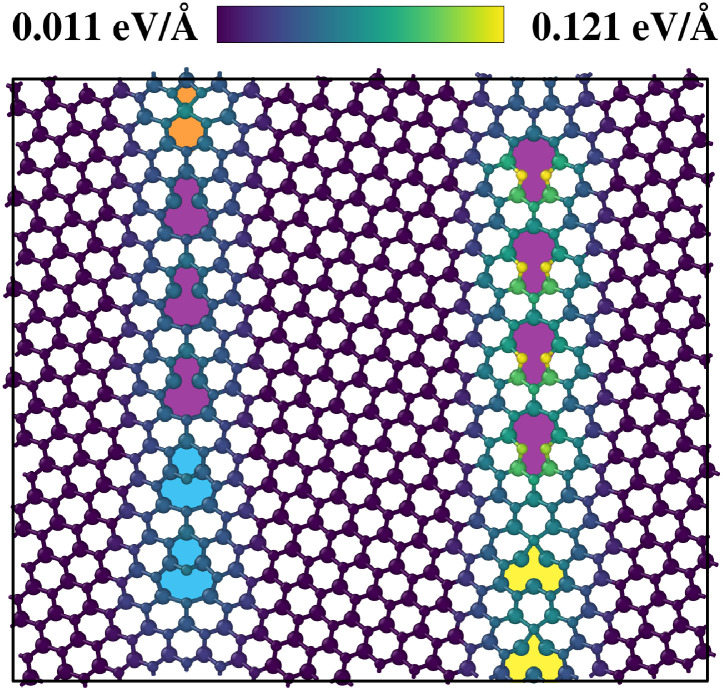
Local uncertainties of grain boundary ring motifs. The color bar represents the local uncertainties *s*^local^_*ij*_ for every atom, calculated by the MACE committee for a MoS_2_ monolayer grain boundary tilted by 18.5°. The cutoff radius for the uncertainty calculation was chosen as *r*_cut_ = 4.0 Å. Motifs such as 4|6 (orange), 5|7 (violet) and 6|8 (cyan) rings are highlighted by coloring. Ten-fold rings also present in the corners of the T_1_ and T_2_ defects are indicated by yellow.

Fig. 9 illustrates the calculated *s*^local^_*ij*_. Considering first the Mo-rich interface on the left side of the structure, it is seen that motifs already present in the threefold rotational defect structures, such as Mo-rich five- and seven-fold rings (5|7), display local uncertainty values almost as low as the pristine environment. More surprisingly, unknown motifs, such as Mo-rich four- and six-fold (4|6) and six- and eight-fold (6|8) rings, do not increase the resulting values in a significant way. To assess the transferability of the force field to grain boundaries, we computed DFT reference forces for the grain boundary structure shown in Fig. 9. An *α*-correlation plot illustrating the relationship between local uncertainties and force errors for this structure is provided in Fig. S6 in the SI. As in previous cases, the local uncertainties are found to be predictive of the corresponding local force errors. This underlines that a CMA-ES run visits a diverse set of structures.^48,49^

The other S-rich interface on the right consists of the same motifs, but here the Mo and S atoms are switched in place (sometimes called opposite polarity). For this reason, two sulfur atoms of the pentagon structure in the S-rich 5|7 rings are placed closer to each other than in the structures known by the force field. This circumstance leads to the increased local uncertainties that are displayed for these specific sulfur atoms.

## Conclusions

We investigated the potential energy landscape of three-fold rotational defects in monolayer MoS_2_ by coupling a machine-learning force field with an evolutionary structure search algorithm. To enhance search efficiency, the algorithm systematically constrained the exploration to a reduced-dimensional space defined by the symmetry operations of the respective defects. We found that these symmetry-guided trajectories maintained lower global uncertainty throughout the search process, led to energetically more favorable structures, and achieved faster convergence. This strategy allowed us to identify several previously unreported defect configurations at a moderate computational cost, thus completing the catalog of rotational defects in MoS_2_.

While machine-learning force fields can already efficiently and transferable parametrize potential energy surfaces, the structure search itself still relies on heuristic algorithms that require extensive manual tuning and struggle to escape local minima. Replacing these heuristics with data-driven approaches capable of learning adaptive search strategies from large datasets presents a promising direction for future work.

## Author contributions

Alexander Adel: conceptualization, investigation, methodology, software, validation, visualization, writing – original draft. Ralf Wanzenböck: conceptualization, methodology, software, supervision, writing – review & editing. Georg K. H. Madsen: conceptualization, methodology, project administration, supervision, writing – review & editing.

## Conflicts of interest

There are no conflicts to declare.

## Supplementary Material

CP-028-D5CP03121D-s001

## Data Availability

Additional figures including parity, correlation, distribution and uncertainty plots have been included as part of the supplementary information (SI). Supplementary information is available. See DOI: https://doi.org/10.1039/d5cp03121d. The trained models and data set splits, together with structure files containing the founders and results presented in this paper, are available on Zenodo at https://doi.org/10.5281/zenodo.16812610. The latest version of Clinamen2, along with its documentation and example evolution scripts, is available at https://github.com/Madsen-s-research-group/Clinamen2-public-releases.
