# Supplementary material for: Completing the hierarchy of rotational defects in monolayer MoS2 through symmetry-aware evolutionary search
Source: Phys Chem Chem Phys. 2025 Dec 18;28(2):1626–33. doi: 10.1039/d5cp03121d (PMC12713517; doi:10.1039/d5cp03121d)
Supplement: CP-028-D5CP03121D-s001 [file CP-028-D5CP03121D-s001.pdf]

Supplementary Information (SI) for Physical Chemistry Chemical Physics.
This journal is © the Owner Societies 2025

Supplementary material for „Completing the Hierarchy of Rotational Defects in Monolayer MoS_2 through Symmetry-Aware Evolutionary Search“

Alexander Adel, Ralf Wanzenböck and Georg K. H. Madsen*
Institute of Materials Chemistry – TU Wien, Austria

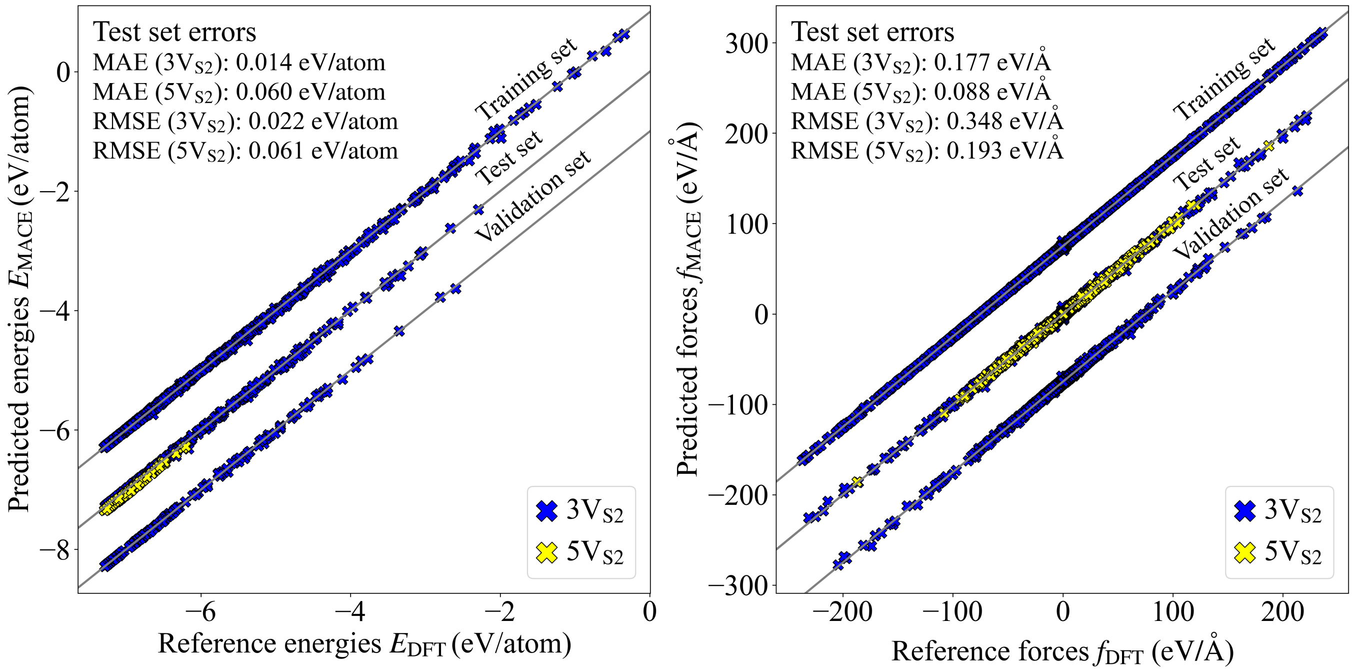

a Parity plots

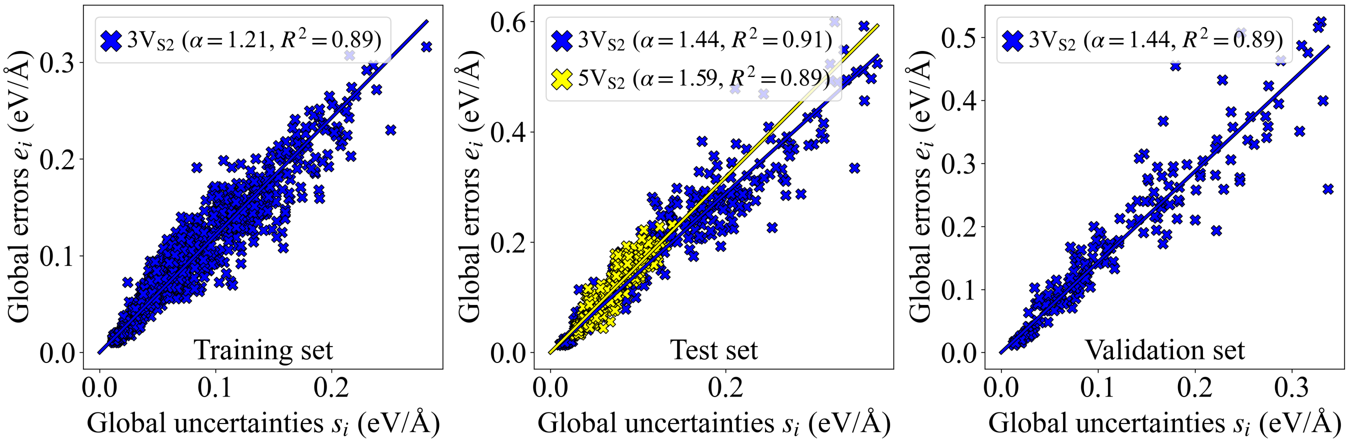

b Alpha plots

Figure S1 – Parity and alpha plots before one active learning step (iteration 0).

*E-mail: georg.madsen@tuwien.ac.at

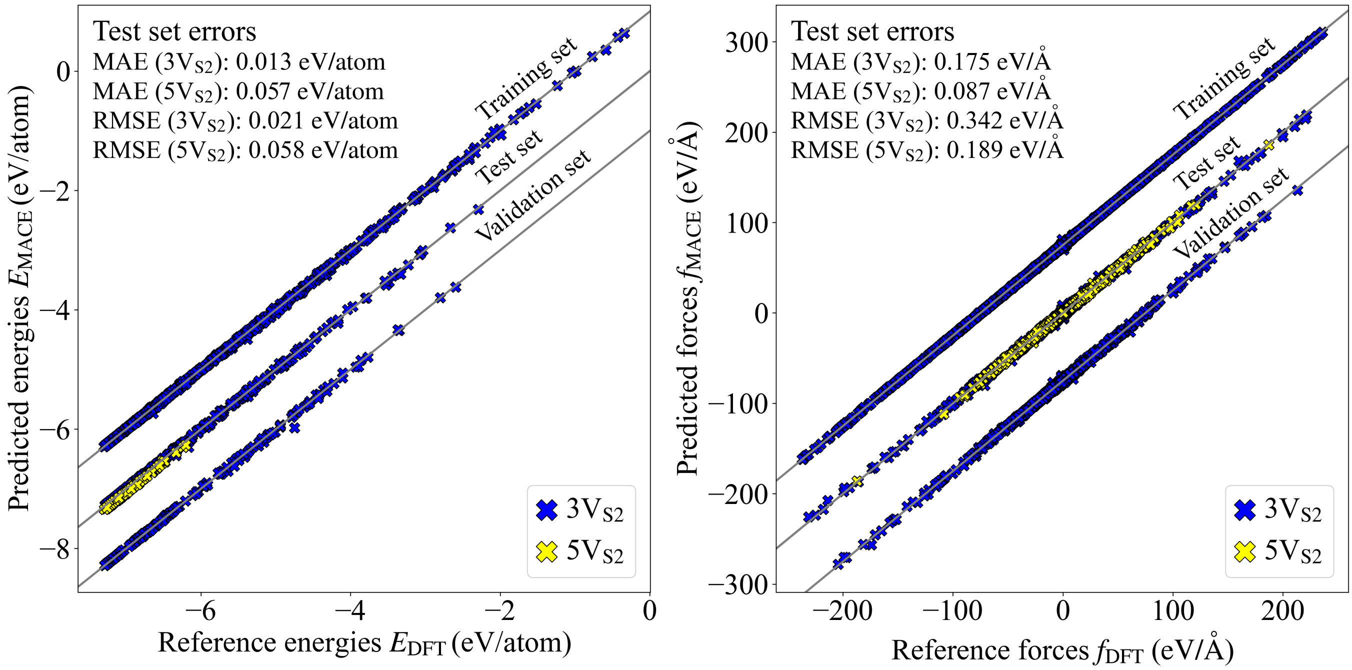

a Parity plots

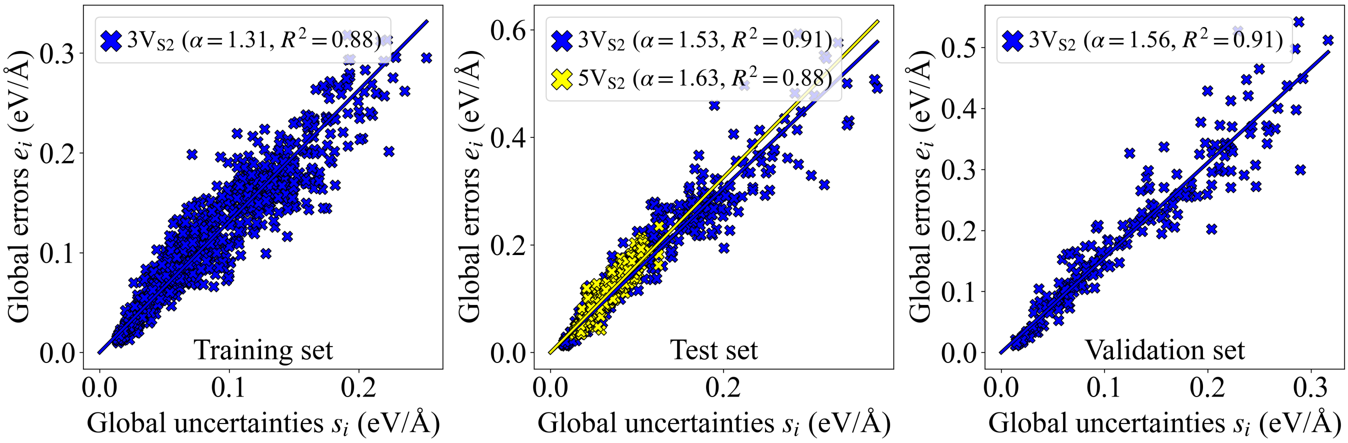

b Alpha plots

Figure S2 – Parity and alpha plots after one active learning step (iteration 1).

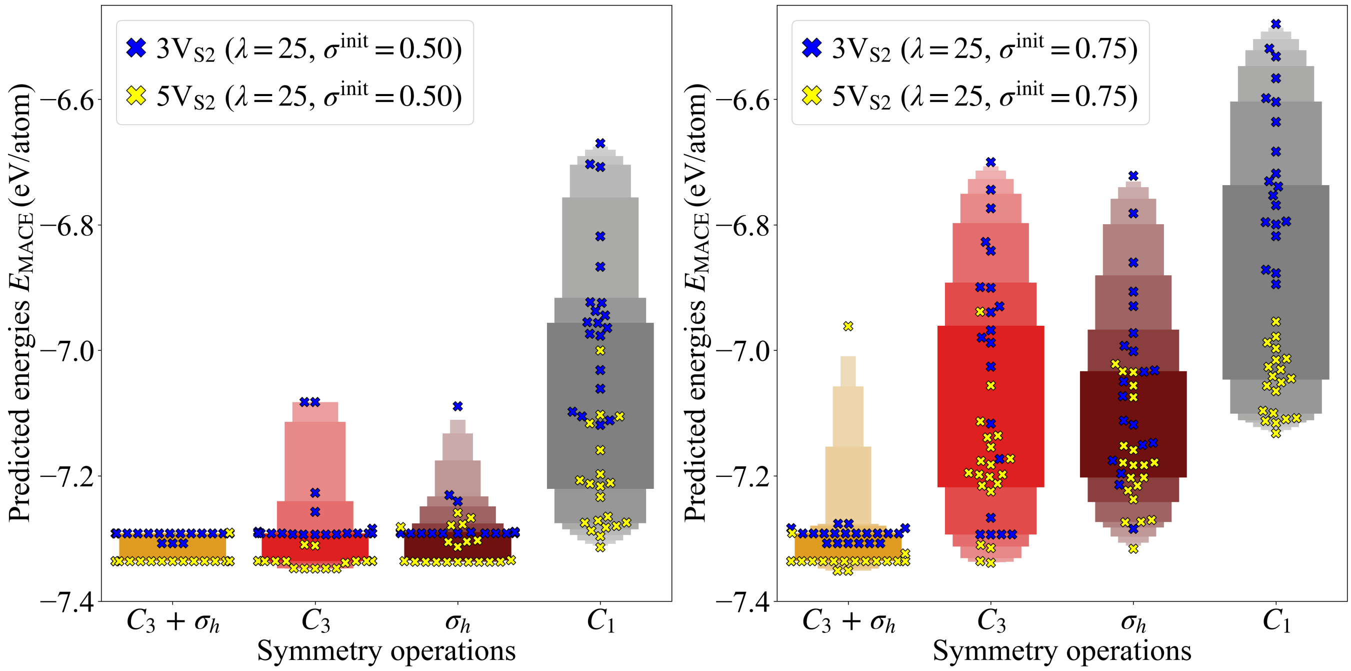

a Population size of 25 individuals

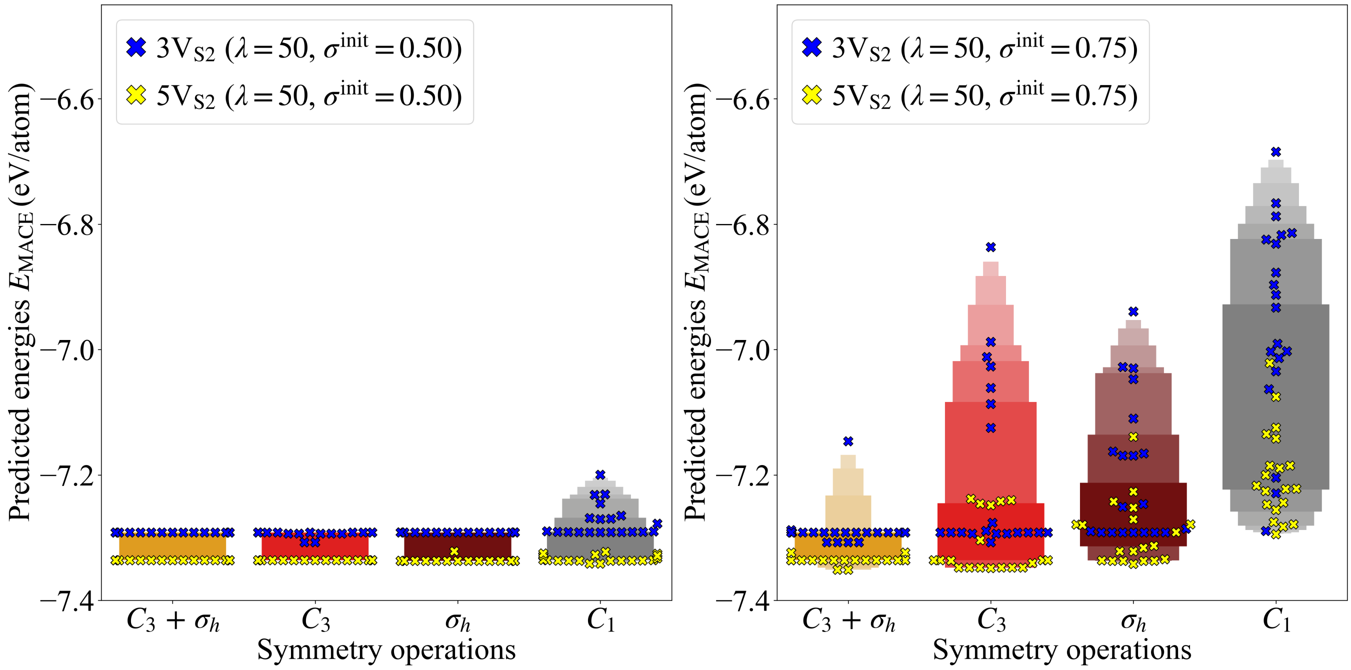

b Population size of 50 individuals

Figure S3 – Predicted energy per atom distributions for all symmetry operations.

Table S1 – DFT calculations of the most stable final symmetry-class structures from the CLINAMEN2 evolutions shown in Fig. 5. **(Upper table)** The MACE and DFT energy differences between the lowest energy and the second lowest energy structures of a given evolution. **(Lower table)** The MACE and DFT energy differences between the lowest energy structures of different symmetries restrictions are given.

Symmetry		Defect	ΔE_{MACE} (eV/atom)	ΔE_{DFT} (eV/atom)
$C_3 + \sigma_h$	1st 2nd	$3V_{S2}$	0.016	0.013
		$5V_{S2}$	0.015	0.020
C_3	1st 2nd	$3V_{S2}$	0.027	0.102
		$5V_{S2}$	0.024	0.038
σ_h	1st 2nd	$3V_{S2}$	0.070	0.072
		$5V_{S2}$	0.042	0.039

Defect			ΔE_{MACE} (eV/atom)	ΔE_{DFT} (eV/atom)
$3V_{S2}$	$C_3 + \sigma_h$	C_3	0.014	0.041
		σ_h	0.023	0.017
$5V_{S2}$	$C_3 + \sigma_h$	C_3	0.013	0.018
		σ_h	0.035	0.033

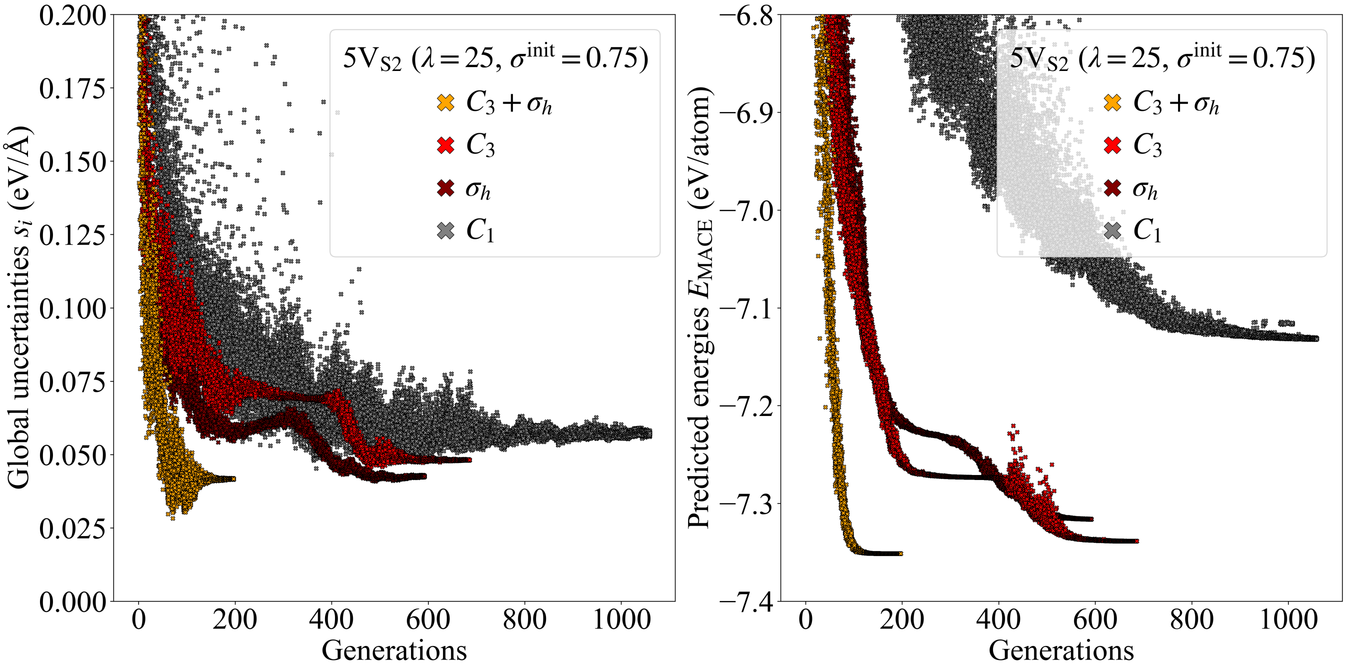

a Selected global uncertainties and predicted energies plotted over generations

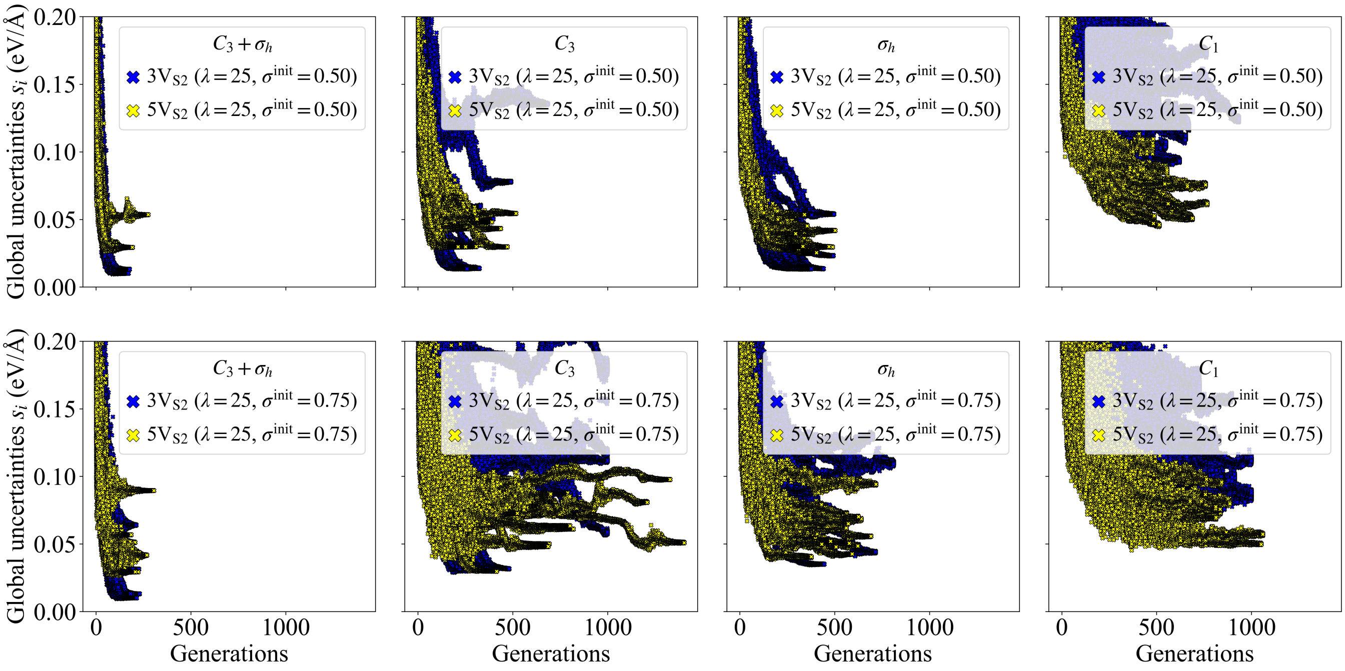

b Global uncertainties plotted over generations

Figure S4 – Global uncertainties and predicted energies plotted over generations.

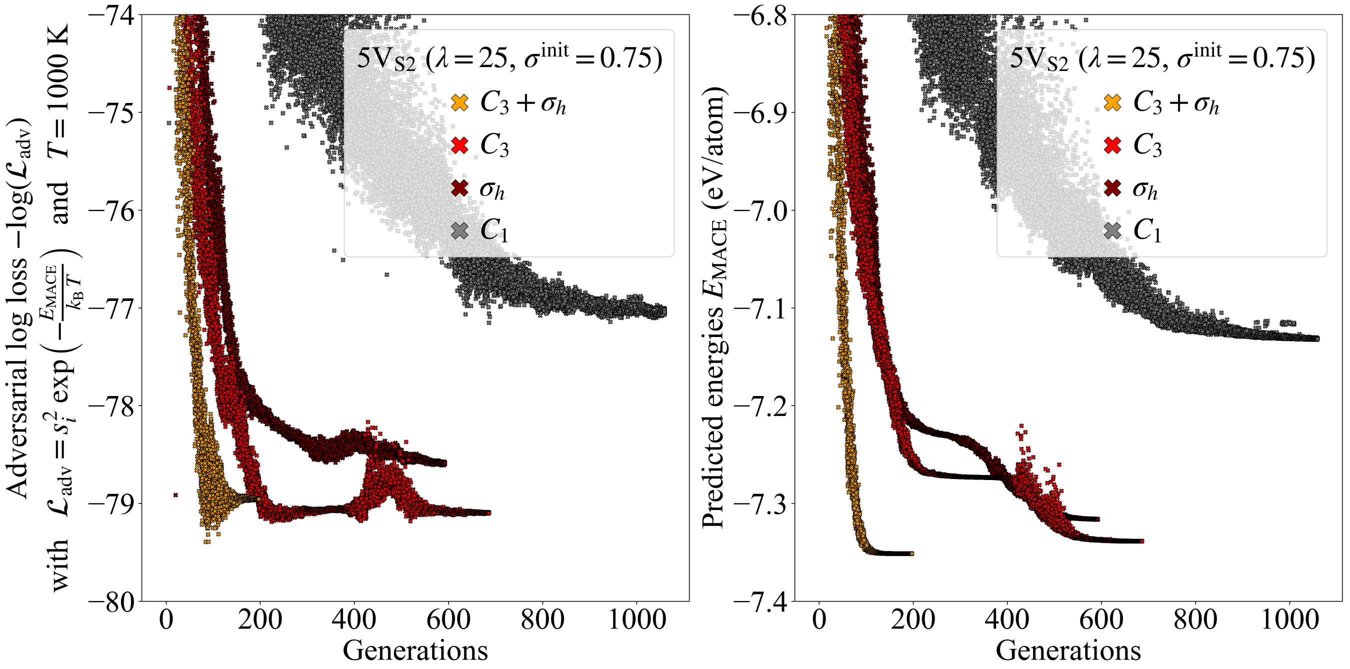

a Selected adversarial log loss and predicted energies plotted over generations

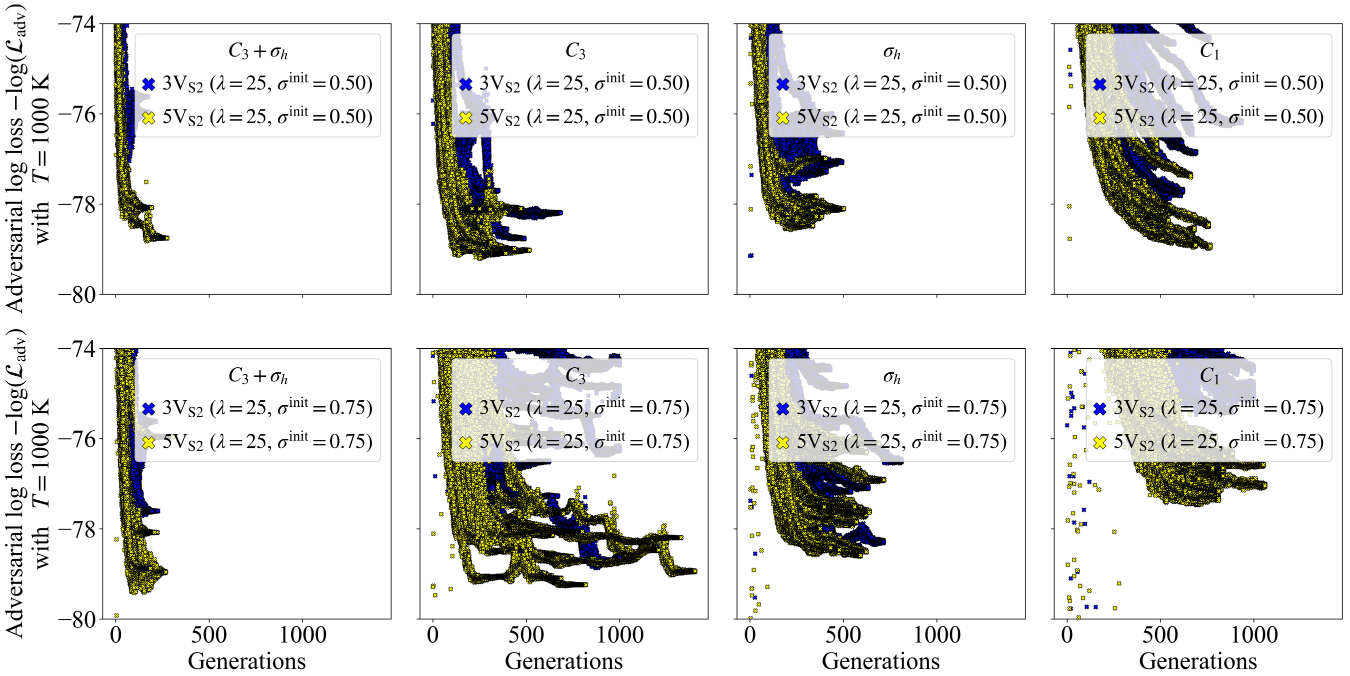

b Adversarial log loss plotted over generations

Figure S5 – Adversarial log loss and predicted energies plotted over generations.

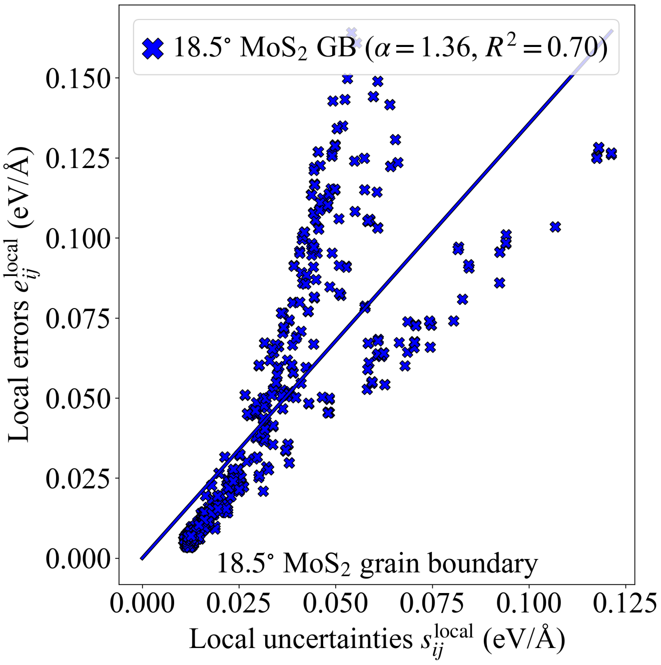

Figure S6 – Alpha correlation plot of the 18.5° MoS_2 monolayer grain boundary structure. Shown are the relations between the local uncertainties s_{ij}^{local} and the local errors e_{ij}^{local} (differences between DFT and MACE forces) for every atom. The cutoff radius for the summation over neighboring atoms was chosen as $r_{\mathrm{cut}} = 4.0\,\mathrm{\AA}$ for both quantities.
